# Multivariate Statistics, Mineralogy, and Radiological Hazards Assessment Due to the Natural Radioactivity Content in Pyroclastic Products from Mt. Etna, Sicily, Southern Italy

**DOI:** 10.3390/ijerph191711040

**Published:** 2022-09-03

**Authors:** Francesco Caridi, Sebastiano Ettore Spoto, Antonio Francesco Mottese, Giuseppe Paladini, Vincenza Crupi, Alberto Belvedere, Santina Marguccio, Maurizio D’Agostino, Giuliana Faggio, Rossella Grillo, Giacomo Messina, Francesco Barreca, Valentina Venuti, Domenico Majolino

**Affiliations:** 1 Dipartimento di Scienze Matematiche e Informatiche, Scienze Fisiche e Scienze della Terra, Università degli Studi di Messina, V.le F. Stagno D’Alcontres 31, 98166 Messina, Italy; 2 Agenzia Regionale per la Protezione dell’Ambiente della Calabria (ARPACal)—Dipartimento di Reggio Calabria, Via Troncovito SNC, 89135 Reggio Calabria, Italy; 3 Dipartimento di Ingegneria dell’Informazione, delle Infrastrutture e dell’Energia Sostenibile (DIIES), Università “Mediterranea”, Loc. Feo di Vito, 89122 Reggio Calabria, Italy

**Keywords:** pyroclastic products, radioactivity, radiological risk, mineralogy, multivariate statistics

## Abstract

In this article, an investigation of the natural radioactivity content of pyroclastic products from Mt. Etna, eastern Sicily, Southern Italy, was carried out. In particular, the assessment of the average activity concentration of the investigated radionuclides, related to the mineralogical phase composition of the analyzed samples, and the radiological health risk for the population, was performed. High Purity Germanium (HPGe) gamma-ray spectrometry was employed in order to quantify the average specific activity of ^226^Ra, ^232^Th, and ^40^K natural radioisotopes. The absorbed gamma dose rate (D), the radium equivalent activity (Ra_eq_), the hazard indices (H_in_ and H_ex_), the annual effective dose equivalent outdoor (AEDE_out_), and the excess lifetime cancer risk (ELCR) were also estimated in order to assess any possible radiological hazard for the population. In our case, they were found to be lower than the maximum recommended values for the population members, thus reasonably excluding radiological hazard effects. Moreover, the identification of the source of the aforementioned naturally occurring radionuclides was attempted by X-ray Diffraction (XRD) and Micro-Raman Scattering (MRS), thereby recognizing the main radioisotope-bearing minerals present in the investigated pyroclastic products. Finally, Pearson correlation, Principal Component Analysis (PCA), and Hierarchical Cluster Analysis (HCA) were performed by processing observed radioactivity and radiological parameters in order to determine their correlation with the sampling locations.

## 1. Introduction

Natural radionuclides from the Earth’s crust and cosmic rays and artificial radioisotopes from nuclear tests and nuclear power plant accidents are always present in the environment and represent significant sources of radiation exposure for humans [1]. Natural radionuclides mainly consist of uranium (^238^U and ^235^U) and thorium (^232^Th) decay chains products and ^40^K, which are primordial and vary in concentrations according to local geological formations [2,3]. Their importance lies in the fact that their annual effective dose rate outdoors accounts for more than half of the radiation exposure to which the public is exposed [4,5]. Therefore, the activity concentrations of radionuclides in environmental matrices are significant for determining background radiation levels in order to evaluate the effects of radiation exposure for human beings [6].

In particular, pyroclastic products, mixtures of rock, mineral, and glass particles expelled during a volcanic eruption, are natural pozzolanic materials available in many volcanic areas [7]. They are considered as natural resources for the production of low-cost and environmentally friendly building materials with acceptable strength and durability characteristics, which can contribute to sustainable development [8,9]. In particular, the partial replacement of Portland cement by pyroclastic products in mortars and concrete, when available, may lead to a cheaper solution [10]. Moreover, in countries with active volcanoes, such as Southern Italy, pyroclastic products could be used to supply nutrients and reduce CO_2_ from the atmosphere [11].

Mt. Etna (eastern Sicily, Southern Italy) is an active volcano built up during the last 540,000 years by the alternate superimposition of lava flows and pyroclastic deposits [12], and its edifice grew over a sedimentary substratum whose thickness is greater than 1.5 km [13]. The source of Mt. Etna’s magmatism is presumably connected with voluminous mantle melting, likely resulting from the suction of asthenosphere material induced by the backward rolling of the subducting Ionian crustal slab [14,15].

In this study, a multidisciplinary approach was developed with the aim of evaluating the natural radioactivity content of pyroclastic products coming from Mt. Etna [16] and sampled in a surrounding area and assessing any possible radiological hazard for the population due to the external exposure to ionizing radiations. In particular, several analytical techniques such as High Purity Germanium (HPGe) gamma-ray spectrometry, X-ray Diffraction (XRD), and Micro-Raman Scattering (MRS) were employed to estimate the radioactivity contents of the investigated samples and to relate them to the mineralogical composition of the investigated pyroclastic products. Moreover, calculations of the absorbed gamma dose rate (D), radium equivalent activity (Ra_eq_), hazard indices (H_in_ and H_ex_), annual effective dose equivalent outdoors (AEDE_out_), and excess lifetime cancer risk (ELCR) were performed in order to assess any possible radiological health risk for the population [17]. This appears to be a crucial task since, on one side, as widely reported in the literature [18], long-term exposure to uranium and thorium has several health effects, such as chronic lung diseases, acute leucopenia, anemia, and necrosis of the mouth. On the other side, radium exposure can cause bone, cranial, and nasal tumors, whereas thorium exposure can be responsible for lung, pancreas, hepatic, bone, and kidney cancers and leukemia. Finally, in order to analyze the radioactivity and radiological parameters in the pyroclastic product samples, multivariate statistical analyses, i.e., Pearson correlation, Principal Component Analysis (PCA), and Hierarchical Cluster Analysis (HCA), were conducted to find out the possible link between the investigated variables [19,20].

## 2. Materials and Methods

### 2.1. Geological Notes and Sampling

Mt. Etna is one of the most active basaltic stratovolcanoes in the world, where the combination of lava flow with explosive phases takes place [21]. Its structure is constituted by several nested strato-volcanoes [15] that, together with other coeval small eruptive centers, grew on a lava plateau of tholeiitic/transitional composition produced by fissural eruptions [22]. The post-plateau volcanic products, emitted through almost continuous eruptive activity, show a composition ranging from picritic and alkali basalt to trachytes, with mugearites and hawaiites as dominant products [23].

Explosive eruptions of magma, which may last from a few days to several weeks, produce ejected pyroclastic products that fall out over the volcano flanks, disrupting transport systems, contaminating the air, and damaging buildings and infrastructures, having a considerable effect on urban communities living close to the volcano and being potentially hazardous to human health [24]. Despite the abundance of pyroclastic products, they are considered a waste material according to the existing laws, thus limiting their use as construction materials [25]. The distribution of pyroclastic products depends on the initial particle size of the fragments, the height of the eruption column, the rate and duration of the eruption, the prevailing wind conditions, the slope and the roughness of the surface upon which the ash is deposited [26,27].

**Figure 1:**
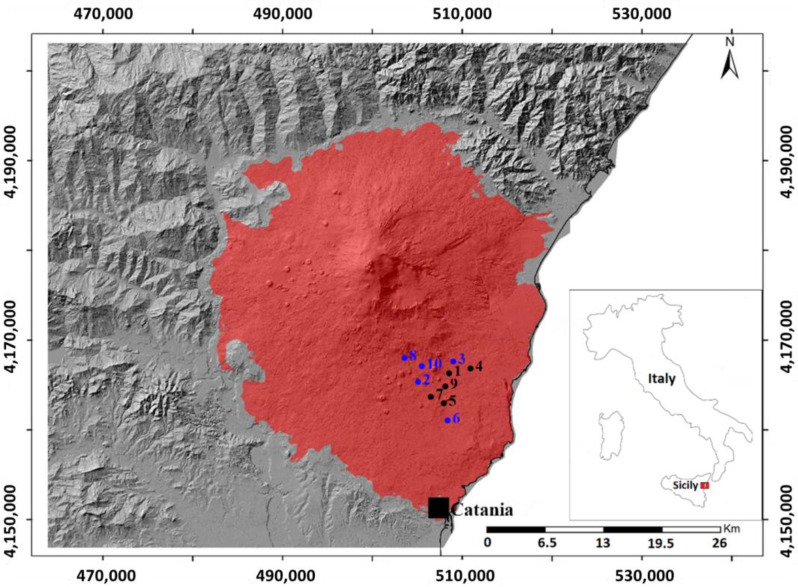
The map of the sampling area. The site IDs of freshly erupted air-fall pyroclastic products (black circle), and of heaps of pyroclastic wastes from previous volcanic activities (blue circle) are reported.

In this scenario, fifty samples of pyroclastic products, around 500 g each, were collected in ten selected sites (ID#, # = 1, …, 10), five samples for each site, of an area surrounding the Mt. Etna volcano (see Table 1 and Figure 1), for subsequent laboratory measurements.

Pyroclastic products were collected during the activity of Mt. Etna that occurred on 21 February 2022. The samples, with sizes ranging from lapilli to ash, were collected according to the protocol for analysis of volcanic ash samples for assessment of hazards from leachable elements [28]. In particular, the sampling was performed from a relatively flat, clean, dry, hard surface, avoiding locations where other surface dust or salts are likely to have accumulated. After the collection, pyroclastic products were stored in clear plastic bags and subsequently transported to the laboratory, avoiding long periods of storage before analysis in order to reduce the impacts of ongoing surface acid reactions with volcanic glass. In five of the selected sites (IDs 1, 4, 5, 7, and 9), freshly erupted air-fall pyroclastic products were collected at the same time or shortly after deposition. In the remaining sites (IDs 2, 3, 6, 8, and 10), samples from heaps of pyroclastic wastes from previous volcanic activities were collected according to the protocol mentioned above [28]. During the sampling of the freshly erupted products, special attention was taken to prevent contact with water either during deposition or after the sampling. Particular precautions were also taken to avoid the contamination of the freshly erupted products with other natural or anthropic particulates.

### 2.2. HPGE Gamma Spectrometry Measurements

For the gamma spectrometry analysis, pyroclastic products were dried until moisture was completely removed, and constant mass was attained. After that, they were inserted into Marinelli hermetically sealed containers of 250 mL capacity in order to be homogeneously distributed around the detector. After 40 days, the secular radioactive equilibrium between ^226^Ra and its daughter products was reached, and the samples were ready for gamma spectrometry counting.

In order to reduce the statistical uncertainty, the samples were counted for 70,000 s, and the spectra were analyzed in order to obtain the specific activity of ^226^Ra, ^232^Th, and ^40^K. In particular, the ^226^Ra activity concentration was calculated by using the 295.21 keV and 351.92 keV ^214^Pb and 1120.29 keV ^214^Bi gamma-ray lines, and the ^232^Th specific activity was determined by using the 911.21 keV and 968.97 keV ^228^Ac γ-ray lines. Finally, for ^40^K, the evaluation was performed from its γ-line at 1460.8 keV.

The experimental set-up was composed of a negative-biased Ortec HPGe detector (GMX), whose operating parameters are reported in Table 2 [29].

The detector was placed inside lead wells to shield the background radiation environment, and a multi-peak Marinelli geometry gamma source (BC-4464) of 250 mL capacity, covering the energy range 60 keV–1836 keV and customized to reproduce the exact geometries of samples in a water-equivalent epoxy resin matrix, was employed for the energy and efficiency calibrations according to the procedure reported in [30].

The activity concentration (Bq kg^−1^ dry weight, d.w.) of the investigated radionuclides was calculated using the following formula [31]:

(1) $$C=\frac{N_{E}}{\varepsilon_{E}t\gamma_{d}M}\;$$

where *N_E_* indicates the net area of a peak at energy *E*, *ε_E_* and *γ_d_* are the efficiency and yield of the photopeak at energy *E*, respectively, *M* is the mass of the sample (kg), and *t* is the live time (s) [32].

The accuracy and repeatability of the results were certified by the Italian Accreditation Body (ACCREDIA) [33].

### 2.3. Assessment of Radiological Hazard Effects

Several radiological parameters, such as the absorbed gamma dose rate (*D*), the radium equivalent activity (*Ra_eq_*), the hazard indices (*H_in_* and *H_ex_*), the annual effective dose equivalent outdoor (*AEDE_out_*), and the excess lifetime cancer risk (*ELCR*), were calculated in order to evaluate the potential radiological hazards and assess the radiation risk to humans.

#### 2.3.1. Absorbed Gamma Dose Rate

The absorbed gamma dose rate calculation is the first major step to evaluate the health risk [34]. This index was calculated as follows [35]:

*D* (nGy h^−1^) = 0.462*C_Ra_* + 0.604*C_Th_* + 0.0417*C_K_* (2)

where *C_Ra_*, *C_Th_*, and *C_K_* are the mean activity concentrations of ^226^Ra, ^232^Th, and ^40^K in the pyroclastic products, respectively.

#### 2.3.2. Radium Equivalent Activity

In order to estimate the gamma radiation dose due to activity concentrations of ^226^Ra, ^232^Th, and ^40^K in the pyroclastic products when used as components of building construction, the radium equivalent activity is regularly utilized. It is an index that describes the activities of ^226^Ra, ^232^Th, and ^40^K in a single activity term [36]:

*Ra_eq_* (Bq kg^−1^) = *C_Ra_* + 1.43*C_Th_* + 0.077*C_K_* (3)

with *C_Ra_*, *C_Th_*, and *C_K_* as the activity concentrations of ^226^Ra, ^232^Th, and ^40^K, respectively. This index is evaluated with conditions that 1 Bq kg^−1^ of ^226^Ra or 1.43 Bq kg^−1^ of ^232^Th or 0.077 Bq kg^−1^ of ^40^K produce an equal gamma dose rate [37].

#### 2.3.3. Hazard Indices

In order to limit the radiation dose to 1 mSv y^−1^, two indices (external and internal radiation hazards) were defined [38]. The internal hazard index (*H_in_*) gives the internal exposure to carcinogenic radon and its short-lived progeny, and it is given by the following formula:

*H_in_* = (*C_Ra_*/185 + *C_Th_*/259 + *C_K_*/4810) ≤ 1 (4)

where *C_Ra_*, *C_Th_*, and *C_K_* are the mean activity concentrations of ^226^Ra, ^232^Th, and ^40^K, respectively. The external hazard index (H_ex_) was calculated using the given equation:

*H_ex_* = (*C_Ra_*/370 + *C_Th_*/259 + *C_K_*/4810) ≤ 1 (5)

Both indices must not exceed the limit of unity for the radiation hazard to be negligible.

#### 2.3.4. The Annual Effective Dose Equivalent Outdoor

The estimated annual effective dose equivalent outdoor received by an individual was calculated using the following equation with an outdoor occupancy of 20% [39]:

*AEDE_out_* (mSv y^−1^) = *D* (nGy h^−1^) × 8760 h × 0.7 Sv Gy^−1^ × 0.2 × 10^−6^ (6)

#### 2.3.5. Excess Lifetime Cancer Risk

The Excess lifetime cancer risk (ELCR) index expresses the probability of developing cancer over a lifetime at a given exposure level. It represents the number of extra cancers expected in a given population as a consequence of exposure to a carcinogen at a given dose and is given by [40]:

*ELCR* = *AEDE_out_* × *D_L_* × *R_F_* (7)

where *AEDE_out_* is the annual effective dose equivalent outdoors, *D_L_* is the average duration of life (estimated to be 70 years), and *R_F_* is the risk factor (Sv^−1^), i.e., fatal cancer risk per Sievert [41]. For stochastic effects, International Commission on Radiological Protection (ICRP) recommends, for this last parameter, a value of 0.05 for the public [41].

### 2.4. Statistical Treatments

Chemometric approaches were conducted using a dedicated statistical software known as XLSTAT (Addinsoft, New York, NY, USA) [42].

In particular, the Principal Component Analysis (PCA) elaboration was run in order to develop an exploratory method useful to reduce the data dimensionality, investigate the correlation degrees among the original variables (Pearson correlation analysis), and identify the principal components (PCs) able to explain the highest values of the sample variability [43]. In order to verify the suitability of the data for factor analysis, the measure of sampling adequacy and Bartlett’s Test of Sphericity were carried out. Both tests’ results (Kaiser–Meyer–Olkin value of 0.722 and chi-square value equal to 37.520 with a statistical significance at *p* < 0.0001) suggested that the correlation matrix was factored and appropriate for Principal Components Analysis. Moreover, the Hierarchical Clusters Analysis (HCA) was also employed to reduce the number of observations, coherently with the Ward’s algorithm, which regroups the samples based on the extent of dissimilarity among them in terms of Euclidian distance [44]. In our case, each group was formed by the samples that show comparable values in terms of radiological parameters and radionuclides activities [45].

### 2.5. XRD Analysis

X-ray diffraction analyses were performed by using a Panalytical Empyrean Diffractometer with Cu K_α_ radiation on a Bragg–Brentano theta–theta goniometer equipped with a solid-state detector, PIXcel [46].

The generator operating parameters were 40 kV and 40 mA. For the measurements, glass slide holders were employed. The 2Θ incidence angle was 5–60°, with a scan speed of 1.2° per minute (continuous scan mode). The total runtime for each analysis was about 45 min.

In order to identify the crystalline mineral components of the investigated samples, COD and RRUFF databases were employed [47].

### 2.6. MRS Analysis

Micro-Raman Scattering (MRS) analyses were performed by using a portable “BTR 111 Mini-RamTM” spectrometer (λ = 785 nm, P_max_ = 280 mW at the excitation port, CCD detector (thermoelectric cooled, TE)) [48,49]. The 65 cm^−1^–3153 cm^−1^ spectral range was investigated, with a resolution of 10 cm^−1^, and an acquisition time of 10 s × 32 scans. The peak at 520.6 cm^−1^ of a silicon chip was used for calibration before each measurement. The system was equipped with a BAC151B Raman microscope. An 80×\40× objective was used, with a working distance of 1.25 mm\3.98 mm and laser beam spot size of 26 µm\50 µm. The maximum power at the samples was ~ 15 mW. Punctual analyses were performed on small sample quantities (about 2 mg). Due to the micrometric size of the grains, a microscopic approach was mandatory in order to select by visual inspection the grains of suspected interest and to that guarantee a good S/N ratio. For each sample, we collected 15–20 spectra from different grains.

For a reliable assignment of the bands, the spectra were compared with the literature [50,51].

## 3. Results and Discussion

### 3.1. The Activity Concentration of the Radionuclides

The average activity concentrations of detected radionuclides, ^226^Ra, ^232^Th, and ^40^K, in the investigated pyroclastic products, are reported in Table 3.

Moreover, some important statistical information (min, max, mean, geometric mean, median, standard deviation, skewness, and kurtosis) of the previous dataset, which is the basic statistic information useful to describe the dataset distribution, is reported in Table 4.

**Figure 2:**
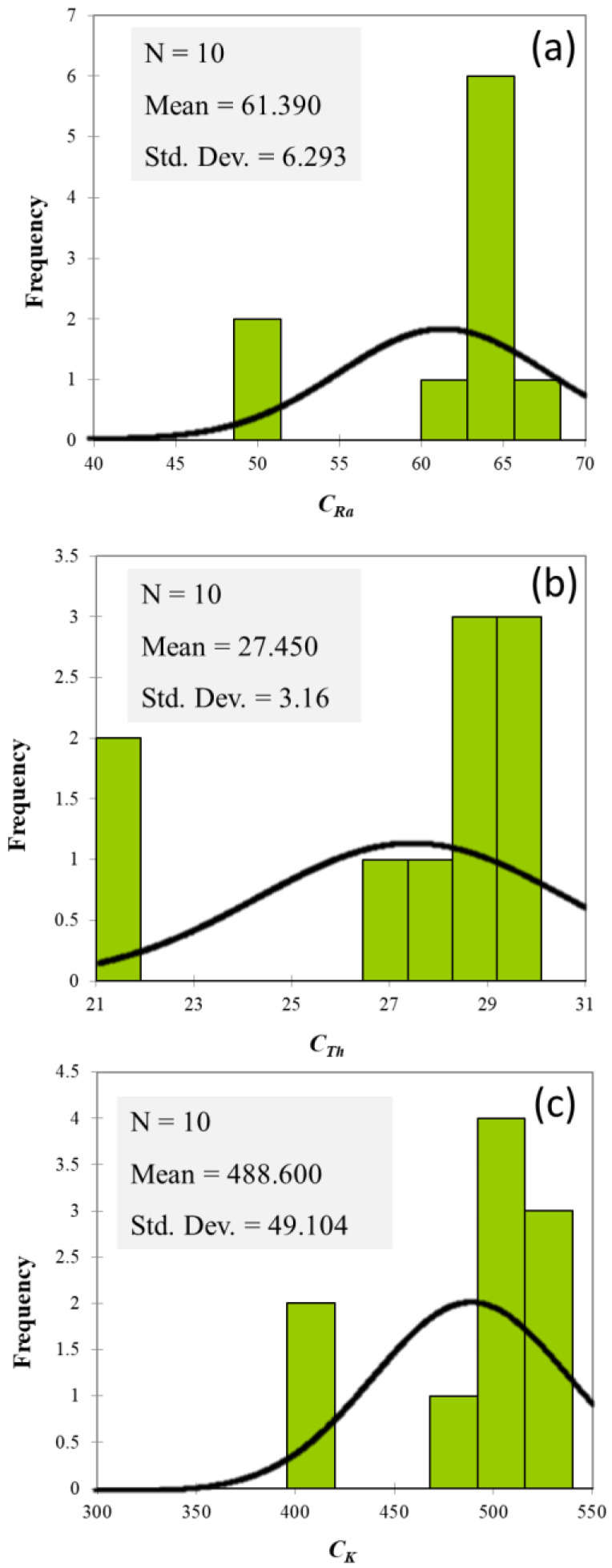
The frequency distributions of ^226^Ra (**a**), ^232^Th (**b**), and ^40^K (**c**) specific activities.

The analysis of natural radionuclide-specific activities was also finalized to build up a model of frequency distributions, as demonstrated by dedicated graphical interfaces (Figure 2).

The highest values of ^226^Ra (67.5 ± 7.5 Bq Kg^−1^ d.w.), ^232^Th (30.0 ± 4.3 Bq Kg^−1^ d.w.), and ^40^K (530 ± 61 Bq Kg^−1^ d.w.) specific activities were detected in the pyroclastic products collected in the Site ID5.

From a statistical point of view, the obtained values of skewness indicate an asymmetric distribution, whereas the kurtosis coefficients confirm how the empirical distribution of ^226^Ra, ^232^Th, and ^40^K, in agreement with the null kurtosis value, can be considered as normal.

Worthy of note, the value of C_Ra_ was higher than the mean value (61.390) in 80% of the samples, whereas in 70% of the pyroclastic products, the ^232^Th and ^40^K specific activities exceed the corresponding average values.

### 3.2. Dose Assessment and Hazard Indices

Table 5 reports the calculated values of the absorbed gamma dose rate together with the radiological hazard indices. The absorbed dose rate was estimated using Equation (2) and values range from 52.4 nGy h^−1^ to 71.4 nGy h^−1^, with an average value of 65.3 nGy h^−1^. The variability of the absorbed doses is attributable to the different lithologic components of the considered areas [52].

The radium equivalent activity (Ra_eq_) was calculated using Equation (3), giving values from 111 Bq kg^−1^ to 151 Bq kg^−1^, with an average value of 138 Bq kg^−1^, lower than 370 Bq kg^−1^, set as the threshold limit for building materials [53]. This indicates that the investigated samples may not be hazardous if used in the field of civil construction. The minimum value of Ra_eq_ was obtained for the pyroclastic products coming from the site, ID8, while the maximum one characterizes the samples collected at the site, ID5.

Going on, the internal and external hazard indices, given by Equations (4) and (5), are lower than unity in all cases, thus excluding radiological health risks.

**Figure 3:**
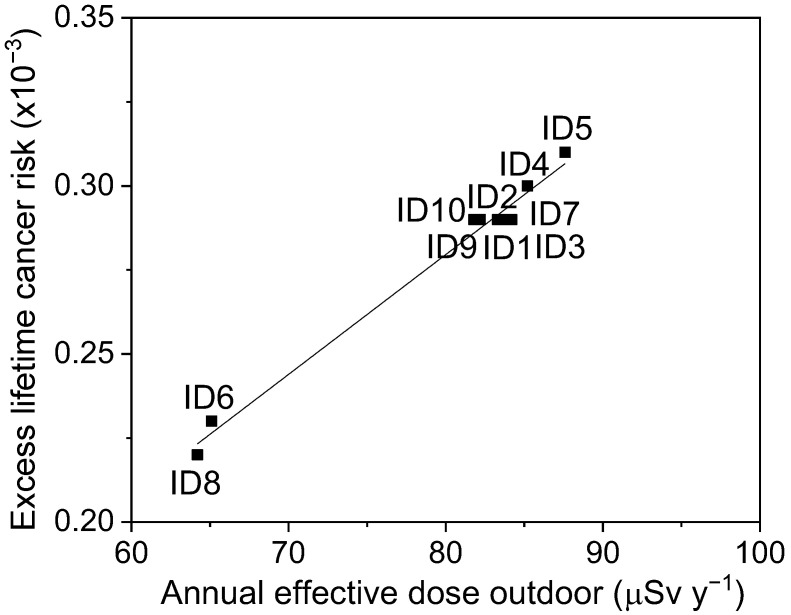
The linear relationship between *ELCR* and the annual effective dose equivalent for all the investigated sampling sites.

Equation (6) was used to evaluate the annual effective dose equivalent outdoors due to the activities of ^226^Ra, ^232^Th, and ^40^K in the analyzed samples. The obtained values range from 64.2 µSv y^−1^ to 87.6 µSv y^−1^, with an average value of 80.2 µSv y^−1^, lower than 1 mSv y^−1^, which is set as the maximum limit by [54]. Worthy of note, a linear relationship between *ELCR*, as calculated by Equation (7), and the annual effective dose equivalent outdoor is found, as shown in Figure 3, is in good agreement with the literature [55]. It is important to underline that the evaluation of the radiological health hazards for the population, only on the basis of the calculated excess lifetime cancer risk, is not possible, since reliable and standardized mortality and morbidity statistics are not accessible.

### 3.3. Statistical Features

As is well established, the determination of a certain distribution of the data plays a key role in parametric statistical elaboration, because if the assumption of the normal distribution is violated, the interpretation of the results could be not valid or reliable. Hence, before proceeding with any relevant statistical procedures, it is fundamental to verify the adequacy of the assumption of the normal distribution of data. For this purpose, Shapiro– Wilk, Anderson–Darling, and Lilliefors tests were run [56].

Table 6 reports the results, in terms of *p*-values, provided by different algorithms used to verify the distribution of data.

The *p*-values results provided by all parametric tests are always lower than the 0.05 value that corresponds to the significant statistic level, confirming the assumption of the normal distribution [57].

The specific activities of detected radionuclides and the radiological parameters were subjected to Pearson’s correlation analysis, in order to put into evidence the interdependency and, at the same time, to assess any existing relationships between the radiological hazard indices and the activity concentrations of the radioisotopes. Table 7 reports the Pearson’s correlation matrix.

The Pearson test shows a significant positive correlation (r = 0.970) between *C_Th_* and *C_Ra_*, for *C_K_* and *C_Ra_* (r = 0.954), as well as for *C_K_* and *C_Th_* (r = 0.925). Furthermore, all detected radiological parameters exhibit a positive strong correlation among them. In more detail, the positive correlations range between 0.968, which indicates the *C_K_*–*H_in_* correspondence between *C_K_* and *H_in_*, and 1.000, obtained for *H_ex_*–*D*, *H_ex_*–*Ra_eq_*, *D*–*Ra_eq_*, *AEDE_out_*–*D*, *AEDE_out_*–*Ra_eq_*, *AEDE_out_*–*H_ex_*, *ELCR*–*D*, *ELCR*–*Ra_eq_*, *ELCR*–*H_ex_*, and *ELCR*–*AEDE_out_* correspondences.

Going on, ten variables (Site IDs, *C_Ra_*, *C_Th_*, *C_K_*, *D*, *Ra_eq_*, *H_in_*, *H_ex_*, *AEDE_out_*, and *ELCR*) were processed by the PCA algorithm. To make the interpretation of the PCA results easier, the Varimax rotation was also performed using the Kaiser normalization procedure [58]. In detail, Table 8 shows the significant factors, extracted before the PCA elaboration, which are the main factors able to explain the variance of the dataset.

**Figure 4:**
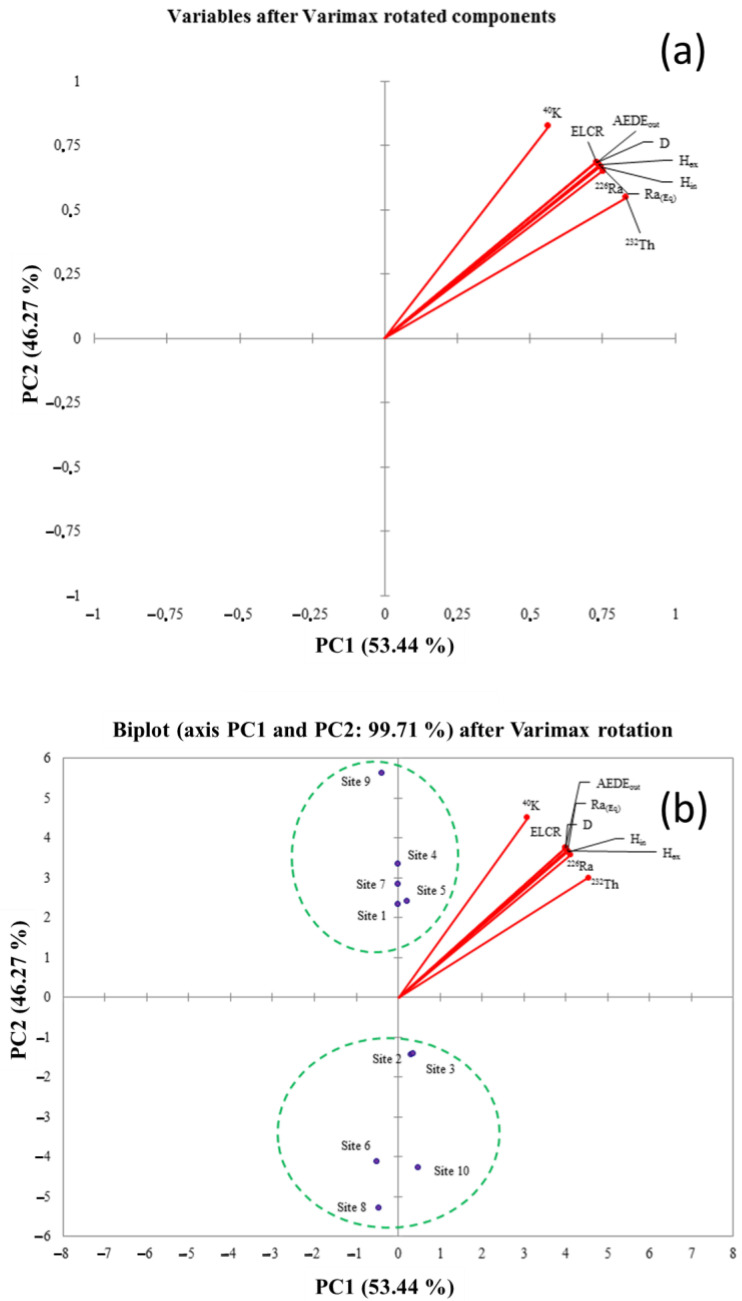
PCA loading plot of the nine original variables (**a**). PCA loading plot with the insert of pyroclastic product sampling sites (**b**).

The PCA and Pearson’s matrix results are coherent, as shown in Figure 4a. In addition, in this figure, the PC1 and PC2 after the Varimax rotation are put into evidence, totally accounting for 99.71% of the total variance. In Figure 4b, the variable “Site IDs” were also inserted, with the attempt to evaluate the possibility of regrouping for those samples that exhibit homogeneous behavior, in terms of activity concentrations and radiological parameters. On that score, the PCA has shown two clusters, the first one composed of ID 1, 4, 5, 7, and 9 sites, whereas the second one was formed by ID 2, 3, 6, 8, and 10 sites. In particular, cluster 1 shows a positive correlation between the analyzed samples coming from ID 1, 4, 5, 7, and 9 sites and all the considered variables. This condition can be graphically appreciated in the PCA plot because cluster 1 and the considered variables fall into the same quadrant. The opposite was found for cluster 2.

Thus, on the basis of the results obtained by the PCA elaboration, it is possible to clearly discriminate two groups of samples. A more critical interpretation will be given in the following, on the basis of the mineralogical composition of the pyroclastic products themselves.

**Figure 5:**
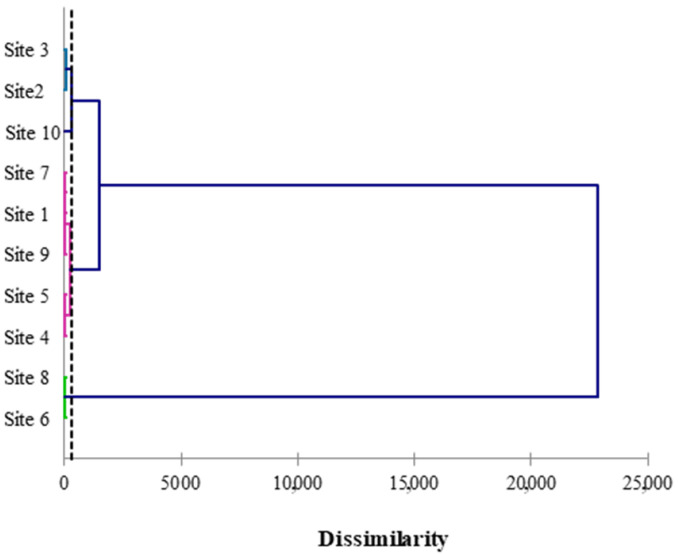
Dendrogram reporting the HCA statistical results.

Finally, with reference to the HCA, the outcome dendrogram is shown in Figure 5.

The dotted line represents the automatic cut, which implies the formation of four clusters, and it was placed on the dendrogram at a 292.11 distance. The first cluster (displayed in a pink color) is more homogeneous and regrouped the ID 1, 4, 5, 7, and 9 sites, whereas the pyroclastic products collected from the ID 2 and 3 sites fall into the second cluster (blue color). Instead, the third cluster was composed of the samples coming from ID 6 and 8 sites (green color), whereas the fourth cluster was formed only by the sample collected in site ID10 (black color).

**Figure 6:**
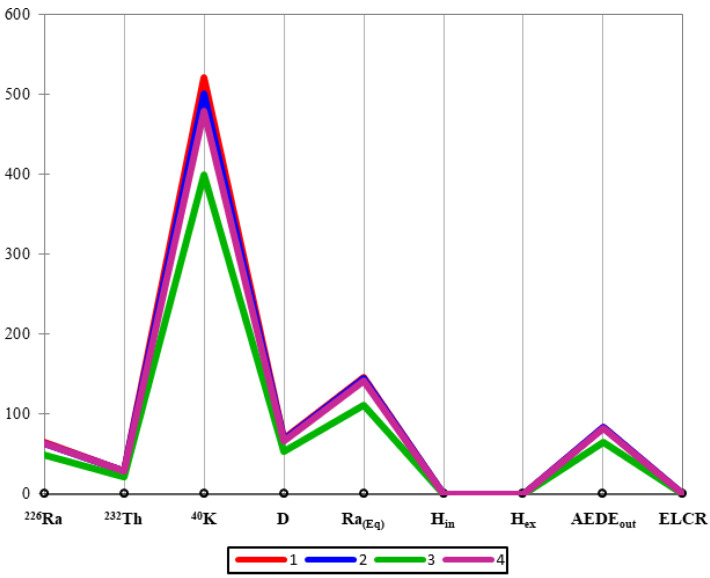
The values of the original variables for the four clusters.

In order to provide information on the discriminating factors used in cluster analysis, the values of the original variables for the four clusters are reported in Figure 6. In detail, the red, blue, green, and purple lines account for clusters 1, 2, 3, and 4, respectively.

Notably, the maximum difference between the reported values was found for ^40^K, *Ra_eq_*, and *AEDE_out_*.

**Figure 7:**
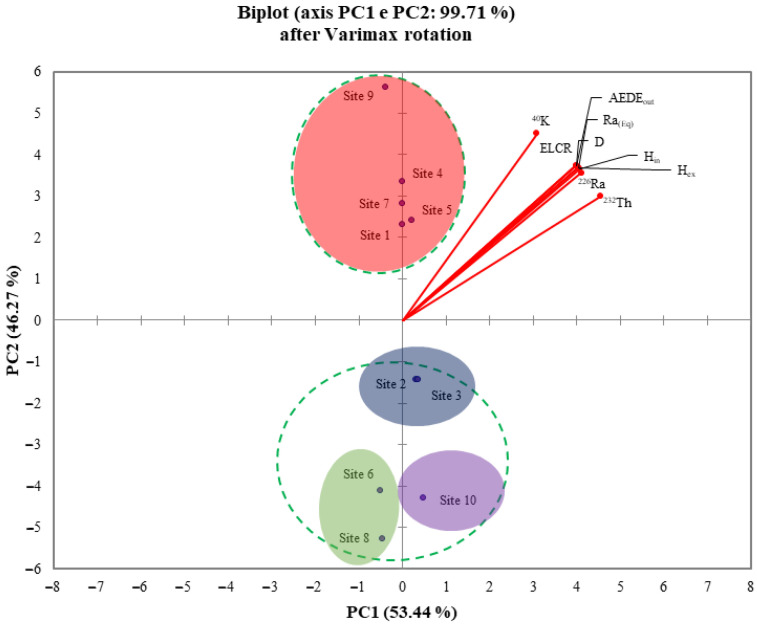
PCA loading plot.

The HCA results are in very good agreement with those obtained after the Pearson’s matrix and PCA elaborations. Moreover, the HCA results increased the degree of detail by grouping in four clusters the site IDs with a good degree of agreement. By carefully observing the site IDs that fall into the respective clusters, it is possible to see how they can be grouped in this way even in the PCA loading plot, as shown in Figure 7.

### 3.4. Mineralogical Composition

Minerals identification was firstly carried out, by matching the measured X-ray Diffraction (XRD) peak positions to the American Mineralogist Crystal Structure Database.

**Figure 8:**
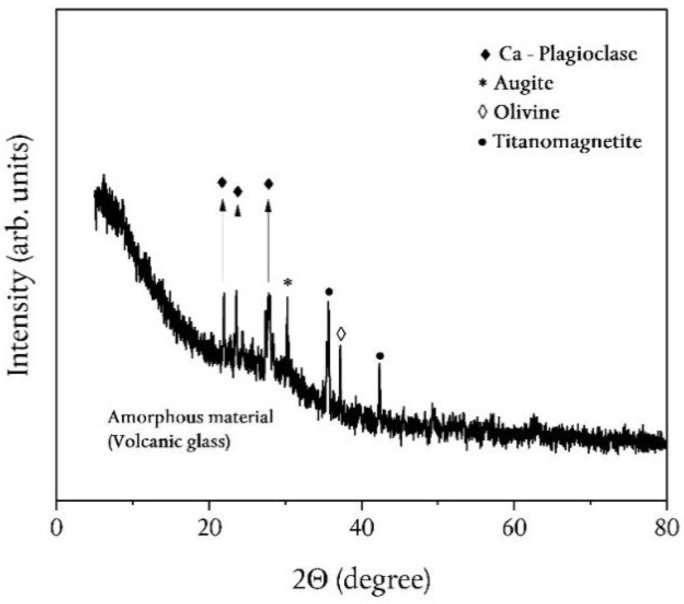
A representative XRD pattern of the sampled pyroclastic products.

The XRD patterns of the investigated pyroclastic products reveal a common assemblage of Ca-plagioclase, Augite (clinopyroxene), Mg-rich–olivine, and Titanomagnetite, superimposed to a glassy groundmass (Figure 8).

Secondly, Micro-Raman Scattering (MRS) was applied in order to support the XRD results.

**Figure 9:**
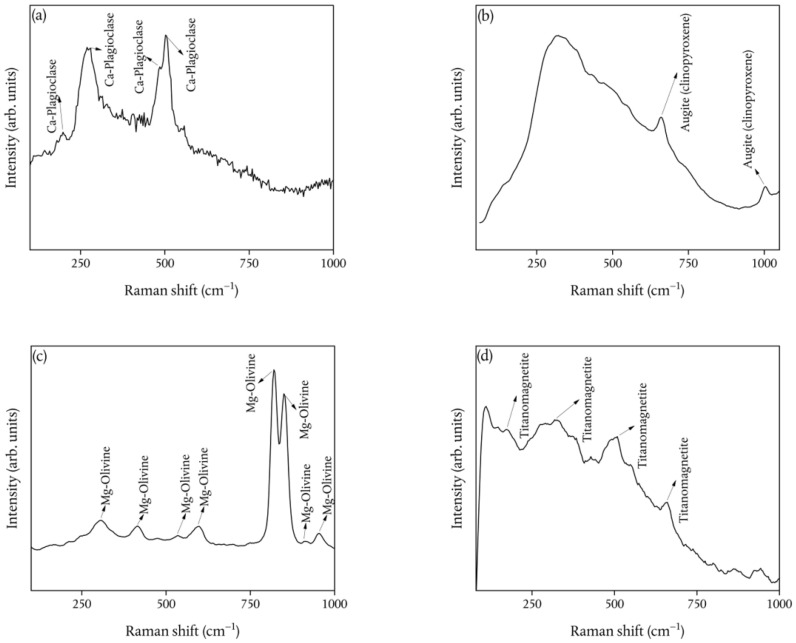
Ca-plagioclase (**a**); Augite (clinopyroxene) (**b**); Mg-olivine (**c**); Titanomagnetite (**d**) Raman spectra, each one being the average of 30 measurements exhibiting similar profiles, collected on different grains of each investigated sample.

Figure 9 shows the Raman spectra collected on different grains of the whole set of investigated samples.

Feldspars are revealed (Figure 9a), as recognized according to [59]. In particular, the two intense Raman peaks in the ~450–520 cm^−1^ range are associated with the ring-breathing modes of the four-membered rings of tetrahedra [60]. Based on a comparison with the literature [59], we can conclude that the feldspars are present as Ca-plagioclases.

Going on, MRS analysis also revealed a second mineralogical phase (Figure 9b), characterized by two Raman bands at ~660 cm^−1^ and at ~1004 cm^−1^. From a comparison with the literature [61], these bands are, respectively assigned to the Si–O bridging stretching and to the Si–O non-bridging stretching of augite (clinopyroxene).

The average spectra reported in Figure 9c exhibit several Raman bands that, according to [62], are ascribed to external SiO_4_ and Mg vibrations (~308 cm^−1^), ν_2_ bending vibrations (~408 cm^−1^), ν_4_ bending vibrations (~ 536 cm^−1^ and ~ 596 cm^−1^), coupled ν_1_– ν_3_ stretching vibrations (~821 cm^−1^ and ~850 cm^−1^), ν_3_ stretching vibrations (~913 cm^−1^ and ~950 cm^−1^), of a Mg-rich–olivine phase.

A less common mineralogical phase is revealed by the Raman spectrum reported in Figure 9d. As a matter of fact, the comparison with the literature data [63] allowed us to recognize the T_2g_ (1) mode (~ 182 cm^−1^), E_g_ mode (~324 cm^−1^), T_2g_ (2) mode (510 cm^−1^), and A_1g_ mode (~660 cm^−1^) of titanomagnetite.

Finally, it is worthy of note that the mineralogical phase composition, as obtained from our samples by XRD and MRS techniques, turns out to be in agreement with that reported in the literature for similar systems [25,64,65].

For the whole set of data reported in this work, we can reasonably retain the detected mineralogical phases and the glassy groundmass responsible for the content of radionuclides previously described. In particular, the activity concentrations reported in Table 3 put in evidence a ^40^K value that appears to be high if compared with the literature data regarding radiological analyses in Etnean volcanic rocks [52].

This can be explained by taking into account that a magmatic system can be described as a liquid silicate melt containing dissolved gas (mainly H_2_O, CO_2_, and S) and variable amounts of crystals [66]. Pyroclastic products are mainly composed of volcanic glass, which is, among volcanic products, the most weatherable component [67]. Freshly deposited pyroclastic products can release high amounts of potentially harmful compounds because adsorbed acidic gases and salts formed by their reaction with the glass are readily soluble [68,69]. In this scenario, radionuclides are vehiculated by volcanic gasses [70].

As underlined in the statistical results, the samples of pyroclastic wastes show different trends from the freshly deposited pyroclastic products. This is explained by the fact that the pyroclastic wastes have undergone contamination and chemical weathering. In this last scenario, according to [71], alkali elements, such as potassium, are more strongly attracted by water than by other inorganic ligands. The same fate is for radionuclides, such as U and Th, that are easily mobilized by dissolution and/or leaching of the pyroclastic products and by devitrification effects [68]. It is important to note how basaltic glass dissolves faster than microcrystalline basalt, according to [72], and the release of alkalis and alkaline earths also varies between glassy and crystalline basalts [72].

## 4. Conclusions

The natural radioactivity content of pyroclastic products coming from the Mt. Etna volcano (Sicily, Southern Italy) was investigated. In particular, calculations of radiological hazard indices were performed in order to assess any possible radiological health risk for the population due to the external exposure to ionizing radiations. In our case, the obtained *D*, *Ra_eq_*, *H_in_*, *H_ex_*, and *AEDE_out_* values were found to be lower than the maximum recommended ones for the population members, thus reasonably excluding radiological hazard effects.

Going on, the mineralogical/geochemical composition of the investigated samples was characterized and correlated with the radioactivity emission. From the results, we can conclude that the investigated pyroclastic products reveal a common assemblage of Ca-plagioclase, Augite (clinopyroxene), Mg-rich olivine, and titanomagnetite. Moreover, the natural radionuclides’ activity concentration reported in this paper put in evidence a high value of ^40^K if compared with the literature data of radiological analyses in Etnean volcanic rocks. This can be explained by taking into account the capability of freshly deposited pyroclastic products of releasing great quantities of potentially harmful compounds. In this scenario, radionuclides are vehiculated by volcanic gasses. At the same time, pyroclastic wastes show different trends from freshly deposited products due to their contamination and chemical weathering.

Finally, multivariate statistical analyses were performed by processing observed radioactivity and radiological parameters in order to determine their correlation with the sampling locations. In particular, the HCA results, in very good agreement with Pearson correlation and PCA, increased the degree of detail by grouping in four clusters the site sampling IDs with a good degree of agreement. Worthy of note, the observed radioactivity and radiological parameters are correlated with the sampling locations following the mineralogical composition of the analyzed samples, i.e., freshly erupted air-fall pyroclastic products in five of the selected sites (IDs 1, 4, 5, 7, and 9), and samples from heaps of pyroclastic wastes from previous volcanic activities in the remaining sites (IDs 2, 3, 6, 8, and 10).

## Figures and Tables

**Table 1:** Site IDs, locations, and GPS coordinates.

Site ID	Sampling Site	GPS Position	
		Latitude	Longitude
1	Zafferana Etnea	37.688641	15.094988
2	Tarderia	37.649748	15.056632
3	Milo	37.71456	15.116151
4	Santa Venerina	37.688089	15.131235
5	Viagrande	37.615743	15.098462
6	S. Giovanni La Punta	37.57984	15.093265
7	Pedara	37.618727	15.056004
8	Rifugio Sapienza	37.698988	15.001205
9	Fleri	37.652492	15.095406
10	Piano del Vescovo	37.699004	15.053896

**Table 2:** The GMX settings.

GMX Detector	
Parameter	Value
FWHM	1.94 keV
Peak/Compton	65:1
ε_r_	37.5% (at the 1.33 MeV ^60^Co γ-line)
ΔV	−4800 V
ΔεE	5 keV–2 MeV

**Table 3:** The average activity concentrations *C_Ra_*_,_ *C_Th_*_,_ and *C_K_* of, respectively, ^226^Ra, ^232^Th, and ^40^K, in the investigated samples.

Site ID	*C_Ra_* (Bq kg^−1^ d.w.)	*C_Th_* (Bq kg^−1^ d.w.)	*C_K_* (Bq kg^−1^ d.w.)
1	63.5 ± 7.2	28.7 ± 3.9	515 ± 60
2	64.6 ± 7.3	28.8 ± 3.9	495 ± 56
3	63.4 ± 7.1	29.9 ± 4.3	506 ± 58
4	64.2 ± 7.1	29.4 ± 4.2	528 ± 62
5	67.5 ± 7.5	30.0 ± 4.3	530 ± 61
6	50.1 ± 5.7	21.7 ± 3.0	404 ± 47
7	65.4 ± 7.3	28.2 ± 3.8	514 ± 58
8	49.4 ± 5.6	21.6 ± 3.1	396 ± 46
9	62.4 ± 7.1	27.2 ± 3.9	519 ± 61
10	63.4 ± 7.1	28.9 ± 4.0	479 ± 55

**Table 4:** Statistical parameters concerning the natural radionuclides activity concentrations.

Statistically Function	*C_Ra_*	*C_Th_*	*C_K_*
Min	49.4	21.6	396
Max	67.5	30.0	530
Mean	61.39	27.45	488.6
Geometric mean	61.07	27.26	486.18
Median	63.45	28.75	510.00
Standard deviation	5.97	3.00	46.58
Skewness	−1.31	−1.26	−1.19
Kurtosis	0.05	−0.08	−0.20

**Table 5:** Radiological hazard indices in the investigated sampling sites.

Site ID	*D* (nGy h^−^^1^)	*Ra_eq_* (Bq kg^−^^1^)	*H_in_*	*H_ex_*	*AEDE_out_* (µSv y^−^^1^)	*ELCR* (×10^−3^)
1	68.1	144	0.56	0.39	83.6	0.29
2	67.9	144	0.56	0.39	83.3	0.29
3	68.5	145	0.56	0.39	84.0	0.29
4	69.4	147	0.57	0.40	85.2	0.30
5	71.4	151	0.59	0.41	87.6	0.31
6	53.1	112	0.44	0.30	65.1	0.23
7	68.7	145	0.57	0.39	84.2	0.29
8	52.4	111	0.43	0.30	64.2	0.22
9	66.9	141	0.55	0.38	82.2	0.29
10	66.7	142	0.55	0.38	81.8	0.29
*Average*	*65.3*	*138*	*0.54*	*0.37*	*80.2*	*0.28*

**Table 6:** *p*-value results of different statistic tests used to verify the normal distribution of data.

Variables	*p*-Value		
	Shapiro–Wilk	Anderson-Darling	Lilliefors
C_Ra_	0.001	0.001	0.000
C_Th_	0.002	0.002	0.015
C_K_	0.006	0.006	0.007

**Table 7:** Pearson’s correlation matrix.

Variables	*C_Ra_*	*C_Th_*	*C_K_*	*D*	*Ra_eq_*	*H_in_*	*H_ex_*	*AEDE_out_*	*ELCR*
***C_Ra_***	**1**								
***C_Th_***	0.970	**1**							
***C_K_***	0.954	0.925	**1**						
***D***	0.994	0.980	0.975	**1**					
***Ra_eq_***	0.994	0.983	0.972	1.000	**1**				
***H_in_***	0.997	0.980	0.968	0.999	0.999	**1**			
***H_ex_***	0.994	0.983	0.972	1.000	1.000	0.999	**1**		
***AEDE_out_***	0.994	0.980	0.975	1.000	1.000	0.999	1.000	**1**	
***ELCR***	0.994	0.980	0.975	1.000	1.000	0.999	1.000	1.000	**1**

**Table 8:** Significant factors, extracted before the PCA elaboration.

	PC1	PC2	PC3
Eigenvalues	8.897	0.077	0.026
Variability (%)	98.855	0.858	0.287
% Total Variance Explained	98.855	99.713	100.000

## Data Availability

Not applicable.
